# The relationship between Central Nervous System morphometry changes and key symptoms in Crohn’s disease

**DOI:** 10.1007/s11682-022-00742-6

**Published:** 2022-11-21

**Authors:** Gita Thapaliya, Sally Eldeghaidy, Michael Asghar, Jordan McGing, Shellie Radford, Susan Francis, Gordon William Moran

**Affiliations:** 1 Division of Child & Adolescent Psychiatry, Department of Psychiatry and Behavioral Sciences, Johns Hopkins University School of Medicine, Baltimore, MD 21287 USA; 2 NIHR Nottingham Biomedical Research Centre, The University of Nottingham, Nottingham University Hospitals NHS Trust and School of Medicine, Nottingham, UK; 3 Sir Peter Mansfield Imaging Centre, School of Physics and Astronomy, The University of Nottingham, Nottingham, UK; 4 School of Biosciences and Future Food Beacon, The University of Nottingham, Nottingham, UK; 5 Translational Medical Sciences Unit, University of Nottingham, Nottingham, UK

**Keywords:** Crohn’s disease, Brain volume, Cortical thickness, Intestinal inflammation, Gut-brain axis, Chronic inflammation, Fatigue

## Abstract

Alterations in grey matter volume (GMV) and cortical thickness (CT) in Crohn’s disease (CD) patients has been previously documented. However, the findings are inconsistent, and not a true representation of CD burden, as only CD patients in remission have been studied thus far. We investigate alterations in brain morphometry in patients with active CD and those in remission, and study relationships between brain structure and key symptoms of fatigue, abdominal pain, and extraintestinal manifestations (EIM). Magnetic Resonance Imaging brain scans were collected in 89 participants; 34 CD participants with active disease, 13 CD participants in remission and 42 healthy controls (HCs); Voxel based morphometry (VBM) assessed GMV and white matter volume (WMV), and surface-based analysis assessed cortical thickness (CT). We show a significant reduction in global cerebrospinal fluid (CSF) volume in CD participants compared with HCs, as well as, a reduction in regional GMV, WMV and CT in the left precentral gyrus (motor cortex), and an increase in GMV in the frontal brain regions in CD compared with HCs. Atrophy of the supplementary motor area (SMA) was associated with greater fatigue in CD. We also show alterations in brain structure in multiple regions in CD associated with abdominal pain and extraintestinal inflammations (EIMs). These brain structural alterations likely reflect neuroplasticity to a chronic systemic inflammatory response, abdominal pain, EIMs and fatigue. These findings will aid our understanding of the cross-linking between chronic inflammation, brain structural changes and key unexplained CD symptomatology like fatigue.

**Supplementary Information:**

The online version contains supplementary material available at 10.1007/s11682-022-00742-6.

## Introduction

Crohn’s disease (CD) patients experience a host of debilitating symptoms, fatigue is a common symptom in active disease, and the second most frequent complaint after extra-intestinal manifestations (EIM) in patients in remission (Singh et al., 2011). Together with abdominal pain and stool frequency (Pariente et al., 2011), fatigue and arthralgia are key variables in the Inflammatory Bowel Disease (IBD) Disability index. These key symptoms are understudied, particularly in relation to their influences on the Central Nervous System (CNS) (Peyrin-Biroulet et al., 2012). Fatigue is mediated via the integration of the CNS and peripheral musculoskeletal systems (Giulio et al., 2006), whereby physiological perturbations occurring in the brain and spinal cord (central fatigue) or at the neuromuscular junction and the skeletal muscle (peripheral fatigue) result in acute and transient decrements in performance. Pro-inflammatory cytokines are involved in symptom generation of central fatigue (Borren et al., 2019), possibly via increasing blood-brain barrier (BBB) permeability, propagating inflammatory signals within the brain via activation of endothelial, glial cells and macrophage, resulting in neuronal cell death (Jones et al., 2006). Systemic inflammation may be linked with demyelinating complications reflected in morphometric changes in the brain of CD patients (Zikou et al., 2014). Intestinal inflammation and abdominal pain may activate central sensitization pathways that convey visceral nociceptive afferent signals from the gut to the brain (Jones et al., 2006; Hubbard et al., 2016), affecting symptom perception and gut function (Jones et al., 2006), with high levels of somatization strongly associated with fatigue severity and impact in inflammatory bowel disease (IBD) patients (Ratnakumaran et al., 2018).

To date, brain morphometry studies relate to CD patients in remission. Using MRI, alterations have been reported in cortical grey matter volume (GMV) (Agostini et al., 2013, 2017; Bao et al., 2015; Erp et al., 2017; Thomann et al., 2020), sub-cortical GMV (Bao et al., 2015; Nair et al., 2016) cortical thickness (CT) (Bao et al., 2015; Nair et al., 2016; Thomann et al., 2016) cortical surface area (CSA) (Nair et al., 2016; Thomann et al., 2016) and cortical folding (Thomann et al., 2016) across multiple brain regions involved in pain, emotion, and homeostasis in CD patients in remission compared to healthy controls (HCs), and GMV has been negatively correlated with disease duration (Agostini et al., 2013; Bao et al., 2015) (see Supplementary Table S1). A recent meta-analysis of voxel based morphometry (VBM) in CD participants in remission showed a significant reduction in GMV in medial frontal gyrus (MFG) compared with HCs (Yeung, 2021). Diffusion Tensor Imaging (DTI) has reported alterations in white matter (WM) fibre integrity in CD patients in remission (Zikou et al., 2014; Hou et al., 2020), suggested to result from cerebral small vessel vasculitis and neurotoxic effects of proinflammatory cytokines (Dolapcioglu & Dolapcioglu, 2015). There are few studies of the relationship of fatigue, abdominal pain and EIM with brain morphometry in CD. CD patients in remission with fatigue are reported to have reduced GMV in left superior frontal gyrus (SFG, a region involved in working memory) compared to HCs without fatigue (du Boisgueheneuc et al., 2006). Abdominal pain in CD participants has been associated with reduced GMV in the insula and anterior cingulate cortex (ACC) compared to CD participants without abdominal pain and HCs (Bao et al., 2017). CD patients with extraintestinal manifestations (EIMs) exhibit altered cortical folding of the ACC and SFG relative to CD without EIMs (Thomann et al., 2016).

This study aims to compare brain morphometry in CD participants with both active disease and in remission with HCs, and to investigate relationships between global/regional GMV, white matter volume (WMV), cerebrospinal fluid (CSF) volume and CT with symptoms of fatigue, abdominal pain, and extraintestinal manifestations (EIM).

## Methods and materials

### Study design

This study was a case-control study of CD participants against HCs., with approval from the National Research Ethics Service [NRES] Committee East Midlands [14/EM/0192], (clinicaltrials.gov [NCT02772458]). CD participants were identified through a clinical database search, expression of interest list and recruited from IBD Clinics at Nottingham University Hospitals. HCs were recruited through participant databases, study fliers and social media. All CD participants and HCs gave informed consent. CD participants disease activity was defined through objective markers of inflammation: recent ileocolonoscopy (Lamb et al., 2019), CT, magnetic resonance enterography [MRE] showing active inflammatory and uncomplicated disease [not stricturing or penetrating behaviour], else FCP > 250 µg/g or CRP > 5 g/dL (Mosli et al., 2015). CD clinical symptoms were measured at inclusion using the Harvey-Bradshaw Index [HBI] score(Harvey, 1980) Stable doses of immunosuppressive agents or biological agents were permitted. Depression and anxiety symptoms were measured using the Hospital Anxiety and Depression Scale (HADS) (Zigmond & Snaith, 1983). *See Supplementary Material for exclusion criteria and additional clinical measures.*

### Image acquisition

Structural brain MRI images were collected as part of a wider MRI protocol including functional MRI brain responses to a test meal. Participants were scanned on a 3T Philips Achieva scanner (Philips Medical Systems, Best, Netherlands) with a 32-channel receive head coil. Brain scans were acquired with a T_1_-weighted MPRAGE sequence orientated along the AC-PC line (1mm^3^,TE/TR = 8.3/3.8ms,flip angle = 8°,SENSE = 2,160 slices,256 × 256 matrix).

### Imaging data analysis

Voxel-based morphometry (VBM) to assess GMV, WMV and CSF volume, and surface-based analysis (SBA) to assess CT were conducted. Preprocessing for both analyses was conducted in CAT12 (Computational Anatomy Toolbox) (version 12.6;http://www.neuro.uni-jena.de/cat/) within SPM12 (version 7771;http://www.fil.ion.ucl.ac.uk/spm/software/spm12/) using MATLAB version 9.7 (R2019b,MathWorks) (See Supplementary Material).

A first level analysis was performed using a general linear model (GLM) implemented in SPM12. An independent t-test was performed to evaluate differences in regional GMV, WMV and CT between CD and HCs, CD with versus without abdominal pain, and CD with versus without EIM. A correlation analysis was performed between IBD fatigue scores, disease duration, HBI and regional GMV, WMV and CT. For VBM analysis, TIV, sex, and age were included as normalized covariates-of-no-interest in the GLM to remove the effects of brain size, sex and age from the data. For CT analysis, age and sex were included as nuisance variables.

Uncorrected analyses were performed at *P* < 0.001, cluster size k > 10, and family wise error (FWE) correction for multiple comparisons was performed with clusters considered significant at *P* < 0.05. Statistical inferences were deduced using nonparametric permutations (5,000) and a Threshold-Free Cluster Enhancement (TFCE) (Smith & Nichols, 2009) correction applied to t-statistic maps (https://www.fil.ion.ucl.ac.uk/spm/ext/#TFCE).

### Non-imaging data analysis

Analysis was performed using SPSS Statistics version 27.0. Variables were tested for normality using a Shapiro-Wilk test. Normal data are expressed as mean ± standard error of mean (SEM) and non-parametric data as median (interquartile range, IQR). Correlation between variables were evaluated using a Spearman for non-parametric data and Pearson’s correlation for parametric data.

## Results

### Participant characteristics

**Fig. 1:**
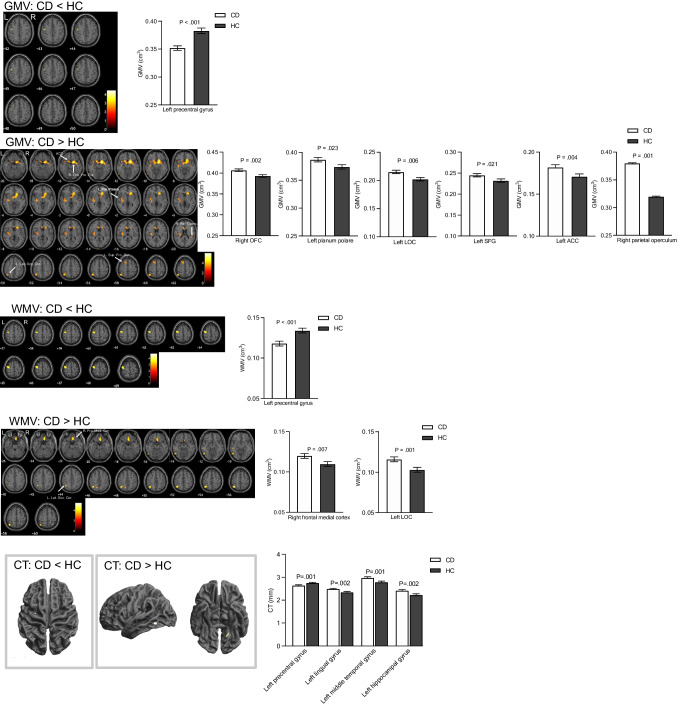
Altered grey matter volume (GMV), white matter volume (WMV) and cortical thickness (CT) in CD compared to HCs. GMV and WMV data assessed using age, TIV and sex, CT data assessed using age and sex as covariates of no interest. All data displayed at *P* < 0.001, uncorrected on a T_1_-weighted normalized anatomical image. OFC = orbital frontal cortex, LOC = Lateral occipital cortex, SFG = Superior frontal gyrus, ACC = anterior cingulate cortex. Note: left superior frontal gyrus, left planum polare, left lateral occipital, and right orbital frontal gyrus survived TFCE FWE corrections (*P* < 0.05) – see Table 1

47 CD participants, 34(72%) with active disease and 13(28%) in remission, and 42 HCs were studied (See Table 2 and S3). A consort diagram of recruitment is shown in Fig. 1. Time between the evaluation of active CD and study visit was 2 (1–8) months. Across all CD participants, age was 31.0(18–68)years (median,range) with a disease duration of 7.5(1–40)years, and C-reactive protein (CRP) of 5(5-224)mg/dl, faecal calprotectin (FCP) 434(18-1800)µg/g, Harvey-Bradshaw Index (HBI) 3(0–9), IBD fatigue score 12.0 (0–15), abdominal pain score 2.0(0–50), TNFα 3.96(0-1234)pg/ml, IL-6 34.8(0-259)pg/ml and IL-1β 1.25(0-1955)pg/ml. HCs were age-matched (30.5(19–65) years). As expected, age and disease duration were significantly positively correlated in CD participants (P = 0.022) and IBDF, abdominal pain, and HBI were significantly intercorrelated (*P* < 0.001). There was no significant difference in circulating cytokines IL-6, IL-1β, or TNF-α serum levels between CD and HC groups. Twelve CD participants had EIMs. No participants had high or severe depression scores, or significant history of psychiatric disorders.

### Altered global structural morphometry in CD compared with HC

No significant difference between CD participants and HCs was found in absolute total intracranial volume (TIV), GMV or WMV, or GMV and WMV when adjusted for TIV alone or TIV, age and sex. A significant reduction in CSF volume was evident in CD compared to HCs (CD:231 ± 4.9 ml (mean ± SEM), HC:258 ± 5.3ml, *P* < 0.001) which persisted after adjusting for TIV, age and sex. No significant difference was found in global CT or age and sex adjusted CT between CD participants and HCs (Supplementary Table S4). Absolute global CT negatively correlated with abdominal pain (Spearman rho=-0.35,p = 0.013) and IBDF (Spearman rho=-0.34,p = 0.034). After controlling for age and sex, correlation between abdominal pain and CT remained significant (P = 0.025), correlation between IBDF and CT was not (P = 0.067). GMV, WMV, and CSF were not associated with fatigue or abdominal pain.

### Altered regional structural morphometry in CD compared with HC

A significant reduction in GMV, WMV and CT was evident in left precentral gyrus in CD compared to HCs. Conversely, CD participants had significantly greater GMV in left lateral occipital cortex (LOC), left superior frontal gyrus (SFG), left planum polare, right orbital frontal cortex (OFC), left ACC and left parietal operculum cortex, as well as greater WMV in right frontal medial cortex and greater CT in the left middle temporal gyrus, left lingual gyrus and left hippocampus in CD compared to HCs. No significant differences were found in any GMV, WMV regions of interest (ROI) between active and remission CD groups before or after adjusting for age, TIV and sex, similarly no significant differences were found in CT (ROI) between active and remission CD groups. Figure 1 shows brain regions with significantly altered regional GMV and WMV, and CT in CD (*n* = 47) compared to HCs (*n* = 42), Table 1.

**Table 1:** Regions showing GMV, WMV and CT alterations in CD participants compared to HCs

	Metric	MNI regions	Hemisphere	MNI coordinates			T value (peak level)	Uncorrected p (peak level)	Uncorrected cluster size	TFCE FWE corrected P	TFE Cluster size
				x	y	z					
CD < HC	GMV	precentral gyrus	L	-46	-9	44	3.46	< 0.001	56		
	WMV	precentral gyrus	L	-48	-10	68	4.73	< 0.001	797		
	CT	precentral gyrus	L	-28	-10	48	3.25	0.001	34		
CD > HC	GMV	superior frontal gyrus	L	-14	14	60	4.15	< 0.001	799	0.035	1012
		planum polare	L	-42	-36	6	4.03	< 0.001	2237	0.048	10
		lateral occipital	L	-44	-62	62	4.6	< 0.001	829	0.04	196
		orbital frontal cortex	R	14	4	-15	5.96	< 0.001	5782	< 0.001	34,316
		anterior cingulate	L	-10	26	12	4.13	< 0.001	444		
		parietal operculum	R	45	-36	21	3.61	<0.001	129		
	WMV	lateral occipital	L	-40	-63	62	5.41	< 0.0001	734		
		frontal medial	R	3	36	-22	3.92	< 0.0001	1069		
		lateral occipital	L	-60	-69	-14	4.18	< 0.0001	272		
	CT	mid temporal gyrus	L	-57	-55	-10	3.5	< 0.0001	75		
		lingual gyrus	L	-19	-59	-7	3.37	0.001	102		
		hippocampus	L	-13	-39	-5	3.26	0.001	32		

### Negative association of fatigue with regional brain volume loss and cortical thinning

**Fig. 2:**
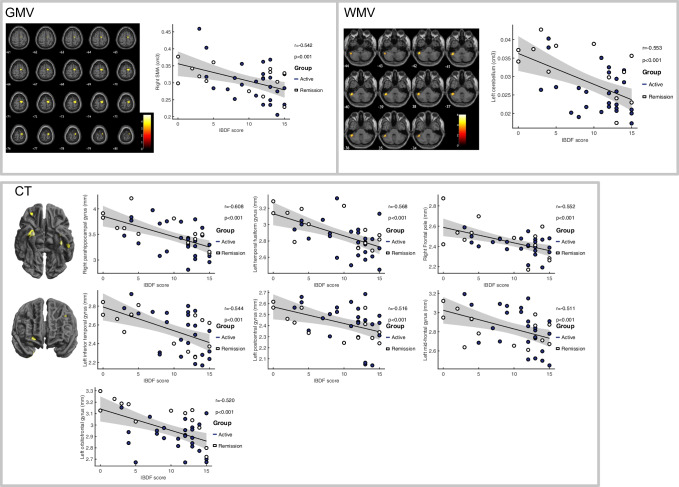
Areas of **a** GMV loss, **b** WMV loss and **c** cortical thinning correlated with increased IBD fatigue score in CD participants displayed on a T_1_-weighted normalized anatomical image (*P* < 0.001, uncorrected), no regions survived TFCE FWE corrections (*P* < 0.05). GMV and WMV data assessed using age, TIV and sex, CT data assessed using age and sex as covariates of no interest. SMA = supplementary motor area

Higher fatigue scores were associated with a reduction in GMV in right supplementary motor area (SMA) as well as in WMV in left cerebellum. Higher fatigue scores were associated with cortical thinning in multiple regions (right para-hippocampal gyrus, frontal pole, left temporal fusiform gyrus, OFC, inferior temporal gyrus, post central gyrus and MFG). Figure 2 shows brain regions with significantly reduced GMV, WMV and CT with greater fatigue in CD (*n* = 39), Table 2.

**Table 2:** Anatomical regions showing an association between GMV and CT with IBD fatigue

Measure	MNI regions	MNI coordinates			Uncorrected p (peak level)	T value (peak level)
		x	y	z		
GMV	Right SMA	10	-12	66	< 0.0001	4.1
WMV	Left cerebellum	-52	-42	-39	< 0.0001	6.01
CT	Right para-hippocampal gyrus	27	-1	-37	< 0.0001	4.54
	Left temporal fusiform gyrus	-38	-21	-25	< 0.0001	4.33
	Right frontal pole	23	53	-5	< 0.0001	4.08
	Left inferior temporal gyrus	-53	-27	-32	< 0.0001	3.81
	Left postcentral gyrus	-12	-38	54	< 0.0001	3.58
	Left midfrontal gyrus	-42	22	39	< 0.0001	3.5
	Right frontal pole	30	43	-13	< 0.0001	3.58
	Left orbitofrontal cortex	-30	30	1	< 0.0001	3.63
	Left temporal fusiform gyrus	-35	-5	-44	< 0.0001	3.38

### Comparison of regional GMV, WMV, and CT between CD participants with and without abdominal pain

**Fig. 3:**
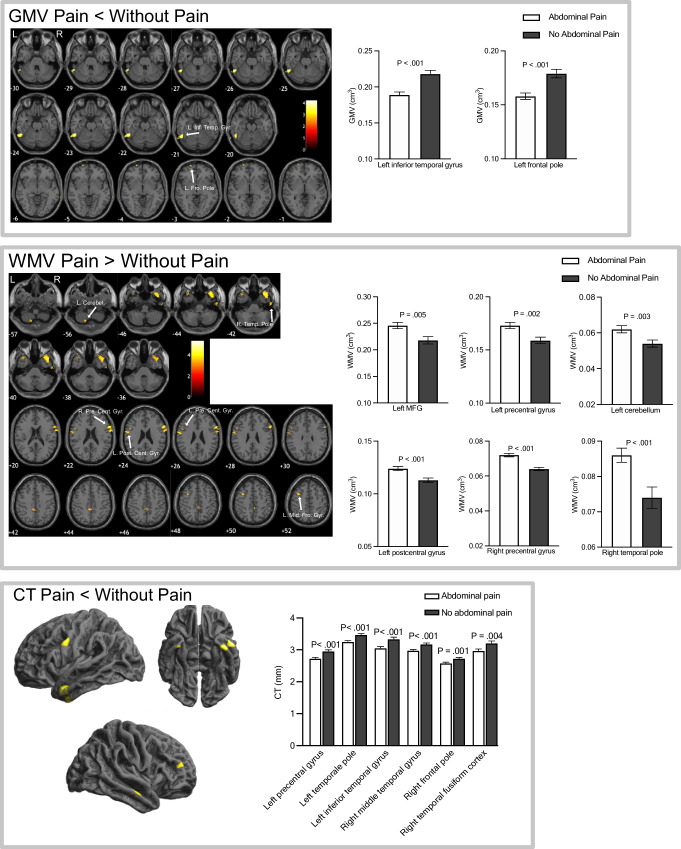
Areas with GMV, WMV and CT alterations in CD participants with abdominal pain compared with CD without abdominal pain. GMV and WMV data assessed using age, TIV and sex, CT data assessed using age and sex as covariates of no interest. Maps displayed on a T_1_-weighted normalized anatomical image at uncorrected *P* < 0.001, no regions survived TFCE FWE corrections (*P* < 0.05)

CD participants with abdominal pain showed a reduction in GMV in left inferior temporal gyrus and frontal pole, as well as cortical thinning in the left precentral gyrus, left temporal pole, left inferior temporal gyrus, right middle temporal gyrus, right frontal pole and right temporal fusiform cortex compared with CD participants without abdominal pain. Conversely, CD participants with abdominal pain showed greater WMV in right temporal pole, right precentral gyrus, left postcentral gyrus, left MFG, left cerebellum and left precentral gyrus compared with CD participants without abdominal pain. There was a significant group effect between pain vs. no pain groups across all GMV, WMV and CT ROIs. Additionally, a group effect between active vs. remission CD groups in the WMV left postcentral gyrus, WMV left precentral gyrus and CT right MFG was present. There were no significant interaction effects between the (CD abdominal pain vs. CD no abdominal pain) (active vs. remission) CD groups, indicating differences observed between the CD abdominal pain group were not influenced by disease status. Figure 3 shows areas with significant alterations in GMV, WMV, and CT in CD participants with (*n* = 27) compared to without abdominal pain (*n* = 20), Table 3.

**Table 3:** Anatomical regions showing GMV, WMV and CT alterations in CD participants with abdominal pain compared with CD without abdominal pain. MFG = middle frontal gyrus

	Measure	Hemisphere	MNI regions	MNI coordinates			Uncorrected p (peak level)	T value (peak level)	Cluster size
				x	y	z			
Abdominal pain < No abdominal pain	GMV	L	inferior temporal gyrus	-63	-54	-30	< 0.0001	4.18	385
		L	frontal pole	-21	74	-9	< 0.0001	4.13	36
	CT	L	precentral gyrus	-56	2	36	< 0.0001	4.1	433
		L	temporal pole	-53	5	-31	< 0.0001	4.68	358
		L	inferior temporal gyrus	-42	-3	-44	< 0.0001	4.07	317
		R	mid temporal gyrus	59	-12	-18	< 0.0001	4	285
		R	frontal pole	40	47	19	< 0.0001	3.57	109
		R	temporal fusiform cortex	34	-4	-44	0.001	3.44	62
Abdominal pain > No abdominal pain	WMV	R	temporal pole	40	21	-48	< 0.0001	5.21	1037
		R	precentral gyrus	60	16	28	< 0.0001	5.31	489
		L	postcentral gyrus	-64	-2	27	< 0.0001	4.84	145
		L	midfrontal gyrus	-38	8	51	< 0.0001	3.79	142
		L	cerebellum	-9	-72	-62	< 0.0001	3.99	126
		L	precentral gyrus	-52	9	26	< 0.0001	3.63	57

### Comparison of regional GMV and CT between CD with and without EIM

**Fig. 4:**
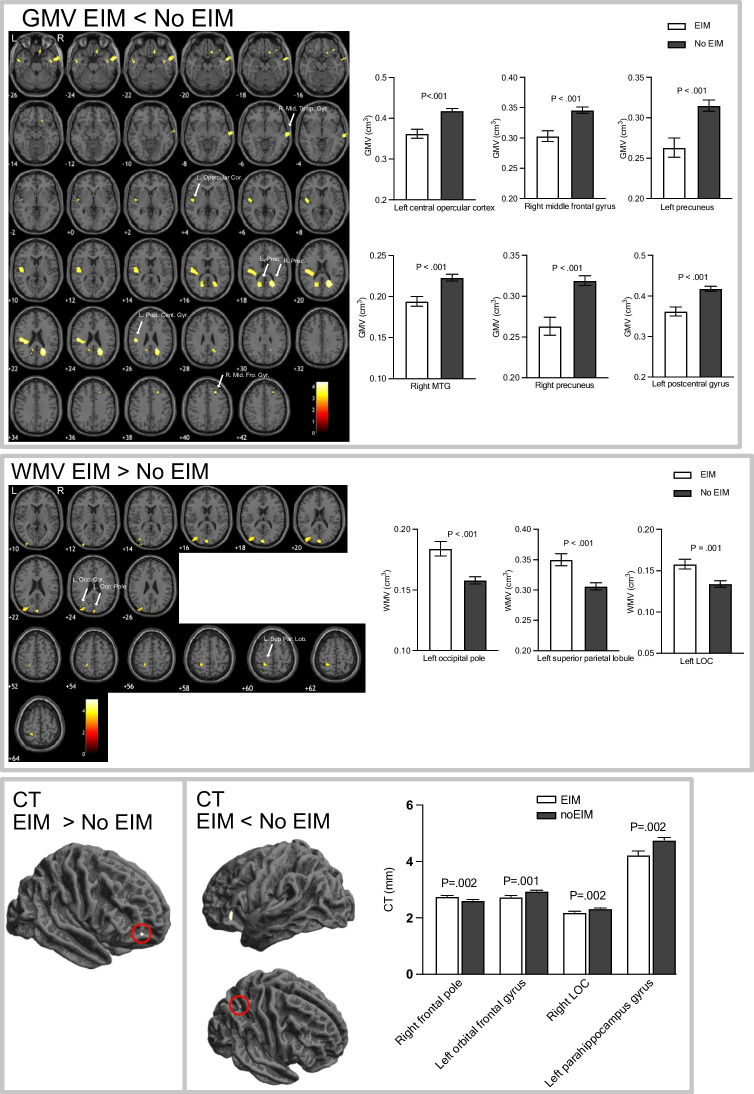
Regions showing alternations in GMV, WMV and CT in CD participants with EIM compared with CD participants without EIM. GMV and WMV data assessed using age, TIV and sex, CT data assessed using age and sex as covariates of no interest. Maps displayed on a T1- weighted normalized anatomical image at uncorrected *P* < 0.001, no regions survived TFCE FWE corrections (*P* < 0.05). MTG = middle temporal gyrus, LOC = lateral occipital cortex, MFG = midfrontal gyrus

CD participants with EIMs had lower GMV in the left postcentral gyrus, left central opercular cortex, bilateral precuneus, right MFG, right middle temporal gyrus, as well as cortical thinning in the left OFC, right LOC and left para-hippocampal gyrus compared to CD participants without EIMs. Conversely, CD participants with EIM had greater WMV in the left LOC, left superior parietal lobule, left occipital pole as well as greater CT in the right frontal pole compared with CD participants without EIM. Figure 4 shows those areas with significant alterations in GMV, WMV and CT in CD participants without EIM (*n* = 35) compared with CD participants with EIM (*n* = 12), Table 4.

**Table 4:** Regions showing alterations in GMV, WMV and CT in CD participants with EIM compared to those without EIM

	Measure	Hemisphere	MNI regions	MNI coordinates			T value (peak level)	Uncorrected p (peak level)	Cluster uncorrected size	TFCE FWE corrected P	Cluster size TFCE corrected
				x	y	z					
EIM < no EIM	GMV	L	postcentral gyrus	-48	-22	26	4.23	< 0.001	1602	0.04	447
		L	central opercular	-48	-10	8	3.83	< 0.001	47	0.049	47
		R	precuneus	27	-60	21	4.36	< 0.001	1113		
		L	precuneus	-20	-63	18	3.83	< 0.001	333		
		R	midfrontal gyrus	30	26	50	3.91	< 0.001	292		
		R	middle temporal gyrus	78	-15	-6	3.74	< 0.001	274		
	CT	L	orbital frontal cortex	-34	29	-3	3.59	< 0.001	134		
		R	lateral occipital cortex	25	-59	49	3.61	< 0.001	32		
		L	Para-hippocampal gyrus	-25	-8	-30	3.35	< 0.001	15		
EIM > no EIM	WMV	L	lateral occipital cortex	-45	-88	20	4.39	< 0.001	542		
		L	superior parietal lobule	-26	-44	60	3.96	< 0.001	249		
		L	occipital pole	0	-93	21	4.6	< 0.001	245		
	CT	R	frontal pole	40	53	-4	3.41	< 0.001	21		

## Discussion

Brain CSF volume was significantly reduced in CD compared with HCs. CSF flow to the brain is essential for protein clearance to prevent accumulation of toxic protein aggregates (Puy et al., 2016). Impaired CSF flow is suggested to result in cognitive deficits in the elderly (Attier-Zmudka et al., 2019). In other systemic inflammatory diseases such as rheumatoid arthritis, pro-inflammatory cytokines in CSF have positively correlated with fatigue (Lampa et al., 2012), and TNF blockade shown to reduce CSF protein levels (Estelius et al., 2019). In chronic fatigue syndrome (CFS), higher fatigue scores associate with reduced CSF volume (Finkelmeyer et al., 2018). The reduced CSF in CD participants may be attributed to systemic inflammation leading to fatigue symptoms. However, we did not show a significant correlation between reduced CSF volume and fatigue scores, or any significant differences in serum cytokine levels between CD and HCs, although prior studies suggest cytokines increase in CSF during systemic inflammation (Engler et al., 2017; Herrick & Tansey, 2021).

Assessment of regional brain volumes showed reduced GMV, WMV and CT in CD participants compared with HCs in left precentral gyrus, the primary motor cortex implicated in motor function, which is in line with previously reported studies in CD (Zikou et al., 2014; Bao et al., 2015). Reduced WMV in the precentral gyrus has been reported in patients with CFS (Finkelmeyer et al., 2018). Atrophy of the left precentral gyrus, evidenced by reduced GMV, WMV and cortical thinning, may be related to fatigue symptoms observed in CD patients. CD patients also showed significantly greater GMV relative to HCs in left SFG, a region implicated in working memory, with alterations likely resulting in cognitive deficits (du Boisgueheneuc et al., 2006). We show increased GMV in the left planum temporale within the superior temporal gyrus (STG), an area implicated in language function (Shapleske et al., 1999) and left ACC in CD compared with HCs. Structural, functional and metabolic alterations in ACC have been reported in CD, and attributed to stress, pain, negative emotions and changes in gut microbiota (Bao et al., 2017; Liu et al., 2018; Lv et al., 2018; Kong et al., 2021, 2022; Li et al., 2021). Further, we show increased GMV in the right parietal operculum, a region implicated in pain (Horing et al., 2019), as well as cortical thickening in the left middle temporal gyrus in CD participants relative to HCs, which is in agreement with Nair et al. (Nair et al., 2016).

Significant alterations were also seen in the sensorimotor network where greater fatigue scores correlated with reduced GMV in right SMA. GM atrophy of SMA may be linked with symptoms of fatigue due to an attenuation of a central drive to peripheral neuromuscular activity. Repetitive transcranial magnetic stimulation of SMA has been shown to increase the recovery rate from central fatigue (Sharples et al., 2016). We show that increased fatigue was also associated with decreased CT in left postcentral gyrus, a somatosensory region, as well as reduced WMV in left cerebellum, a region involved in sensory-motor processing, cognitive and emotional functioning (Schmahmann, 2019), also implicated in chronic fatigue syndrome (CFS) (Barnden et al., 2011). Further, we show a negative correlation between fatigue and CT in right para-hippocampal gyrus, a region showing reduced functional connectivity with greater fatigue scores in CFS patients (Boissoneault et al., 2016). Increase in fatigue also associated with reduced CT in left temporal fusiform gyrus and left inferior temporal gyrus, right frontal pole (anterior part of prefrontal cortex), left MFG and left orbitofrontal cortex (OFC). In patients with CFS, frontal regions are related to attentional resources (i.e. exertion of extra mental effort to improve task performance) (Mizuno et al., 2015). Atrophy of frontal regions associated with increased fatigue may result in attentional deficits and cognitive fatigue leading to enhanced perception of fatigue in CD.

A decrease in global CT was associated with an increase in abdominal pain. Further, CD with abdominal pain had reduced regional GMV in left inferior temporal gyrus and left frontal pole compared to CD without abdominal pain. Cortical thinning was found in CD with abdominal pain compared with those without in temporal regions, left precentral gyrus and the right frontal pole, in contrast to regions previously reported by Bao et al. (Bao et al., 2017). Notably, GMV alterations implicated in pain processing are not solely limited to regions of the pain matrix, (Smallwood et al., 2013; Torta et al., 2014)., with controversy regarding the direction of change (increase or decrease) (Smallwood et al., 2013; Torta et al., 2014). Brain structural alterations associated with pain maybe linked to an imbalance in neurotransmitters (Lv et al., 2018), ongoing nociceptive inputs, heightened attention to nociceptive and unpleasant sensory stimuli leading to use-dependent plasticity effects (May, 2008; Pomares et al., 2017).

The presence of EIM in CD is an indicator of greater inflammatory burden and systemic disease. CD participants with EIMs had reduced GMV in sensorimotor regions of left postcentral gyrus, left central operculum and bilateral precuneus, right middle frontal and right middle temporal gyrus, as well as cortical thinning in the left orbital frontal gyrus, right LOC compared with CD without EIM. We also show greater WMV and CT in the left occipital regions and right frontal pole respectively. Our findings are in contrast to a previous study examining brain structure in relation to EIMs in CD, where no difference in CT was reported (Thomann et al., 2016). EIM-associated brain structural alterations are possibly linked to a chronic inflammatory response and disease burden.

This study has some limitations. There is a variation in disease duration and severity of inflammation and medication use across the CD group. The cross-sectional nature of this study means chronic symptoms are only assessed at a single time point, longitudinal studies are warranted to assess the time course of brain structural changes in CD. Our structural differences may represent neural correlates of different disease courses (e.g. mild vs. complicated), however we were underpowered to study brain structural differences based on disease course.

## Conclusion

This is the largest study to date in patients with active CD. We show a significant reduction in global CSF volume, and regional GMV, WMV and CT in the motor cortex, and an increase in GMV in frontal brain regions in CD compared with HCs. Alterations in brain structure in multiple regions in CD associated with fatigue, abdominal pain and EIMs, may reflect neuroplasticity effects to a chronic systemic inflammatory response and chronic symptom stimuli, explaining the persistence of fatigue symptoms in CD patients in remission.

## Supplementary Information

Below is the link to the electronic supplementary material.

ESM 1 (DOCX 139 KB)

## Data Availability

The datasets used and/or analysed during the current study are available from the corresponding author on reasonable request.
