# Supplementary material for: The relationship between Central Nervous System morphometry changes and key symptoms in Crohn’s disease
Source: Brain Imaging Behav. 2022 Nov 21;17(2):149–60. doi: 10.1007/s11682-022-00742-6 (PMC10049962; doi:10.1007/s11682-022-00742-6)
Supplement: Supplementary file 1 — (DOCX 139 KB) [file 11682_2022_742_MOESM1_ESM.docx]

**Supplementary Materials**

**Table S1**: Overview of MRI studies assessing brain morphometry in CD patients in remission. N/A: not available

| **Study** | **Population** | **Summary Findings** | **Correlation with clinical scores** |
| --- | --- | --- | --- |
| (Bao *et al.*, 2017) | 21 CD with abdominal pain  26 CD without abdominal pain  30 HCs | **GMV:** **CD with pain < CD without pain and HC** in the insula and ACC [ P<0.05, FDR corrected]  **GMV: CD without pain > CD with pain and HC** in HIPP/PHG [ P<0.05, FDR corrected]  **CD with/without pain >HC in putamen** [ P<0.05, FDR corrected] | **GMV** of insula and ACC significantly negatively correlated with daily pain scores in patients with abdominal pain  P < 0.005 (uncorrected), then FDR corrected using small volume correction |
| (Agostini *et al.*, 2017) | 17 CD  17 HCs | **GMV:** No difference between HC and CD | N/A |
| (Erp *et al.*, 2017) | 20 CD with fatigue  17 HCs | **GMV:** **CD<HC** GMV in left superior frontal gyrus P-value < 0.05, corrected for multiple comparison | N/A |
| (Thomann *et al.*, 2016) | 15 CD (9 with EIM)  6 CD with no EIM  15 HCs | **CT:** No differences in CT  **Surface area**: **CD with EIM>HC** left rostral middle frontal gyrus  **Gyrification index (GI): CD>HC** CD with EIM subgroup showed elevated local GI in left lingual gyrus, extending to precuneus, cuneus, PHG and isthmus cingulate  **CD with EIM vs. CD without EIM:** elevated GI in right rostral ACC, extending to superior frontal gyrus and lower GI in the right insula**.** | No alterations were significantly correlated with disease duration |
| (Nair *et al.*, 2016) | 19 CD  20 HC | **CT: CD<HC** middle and superior frontal, superior temporal, superior parietal, precuneus, and pericalcarine regions. **(P<0.001, uncorrected).**  **CT: CD>HC** in left superior frontal region **(P<0.05, corrected),** lateral occipital, post-central, and the superior and inferior temporal regions **(P<0.001, uncorrected).**  **CT: CD>HC** in the right middle temporal, post-central, lateral occipital, and inferior parietal regions (P<0.001, uncorrected). **Subcortical volume**: **CD<HC** Left putamen, left pallidum, right putamen, right pallidum **(P<0.05, uncorrected)**  **Cortical surface area:**  **CD<HC** left precuneus, pars opercularis, fusiform, paracentral, superior temporal and caudal middle frontal and insular regions **(P=0.09, uncorrected)**  **Cortical surface area:** **CD> HC** temporal pole, superior frontal regions **(P=0.09, uncorrected)** | Surface area of the left middle temporal region correlated negatively with VF test.  The VF test was performed outside the scanner, using Controlled Oral Word Association Test, which measured the spontaneous production of words beginning with ‘F,’ ‘A,’ and ‘S’ in three respective 1-min trials. |
| (Bao *et al.*, 2015) | 44 CD remission and 33 HC | **GMV: CD<HC** bilateral ACC, SMA, insula, postcentral gyrus, precentral gyrus, superior frontal cortex, dorsomedial [dmPFC], middle frontal cortex, superior temporal cortex, right MCC, left middle OFC, inferior frontal cortex, middle temporal cortex, inferior temporal cortex, and inferior parietal cortex. [p < 0.05, FDR corrected].  **GMV: CD>HC** periaqueductal grey [PAG], bilateral putamen, left pallidum, HIPP, thalamus, precuneus, posterior parietal cortex [PPC], right amygdala, and cerebellum. [p < 0.05, FDR corrected]  **CT: CD<HC** bilateral OFC, superior frontal cortex, inferior parietal cortex, superior temporal cortex, left insula, rostral [rACC], caudal middle frontal cortex, pars triangularis, postcentral gyrus, middle temporal cortex, PHG, right [PCC], precentral gyrus, and fusiform gyru**s [p < 0.05, corrected]**  **CT: CD>HC none** | Disease duration negatively correlated with GMV of the right ACC, dmPFC, and left insula.  Disease duration negatively correlated with the CT of the left insula and OFC |
| (Zikou *et al.*, 2014) | 18 IBD (11 CD and 7 UC),  20 HC | **GMV: CD<HC** right fusiform, left inferior temporal, left fusiform, right inferior temporal, right precentral, right SMA, right middle frontal, left superior parietal. [P<0.05, FWE, corrected]  **GMV: CD>HC** none |  |
| (Agostini *et al.*, 2013) | 18 CD in remission and 18 HC | **GMV: CD<HC** middle and superior frontal gyrus,  and**. (P<0.001, uncorrected)** after ROI approach SVC in the anterior MCC  **GMV: CD>HC** none | Disease duration negatively correlated with GMV in the left precentral gyrus, RSFG, left/right middle frontal gyrus, and superior frontal gyrus, left subgenual anterior cingulate, right posterior midcingulate, left PCC, left/right PHG, right superior temporal gyrus, left inferior temporal gyrus, [P<0.001, uncorrected] |
| (Yeung, 2020) | ﻿﻿Sixteen original studies comprised of a total of 865 participants, where CD patients in remission (n=486) and HCs (n=379) were meta-analyzed | ﻿**GMV: CD<HC** medial frontal gyrus |  |
| (Thomann *et al.*, 2021) | 31 CD patients in remission, 15 UC patients in remission and 17 HCs | **GMV: IBD<HC**, in precuneus, inferior parietal lobule, superior parietal lobule, middle frontal gyrus, middle temporal gyrus, superior temporal gyrus | ﻿No associations were found between the joint component and clinical parameters including cognitive function (p = 0.72) anxiety (p = 0.53), depression (p = 0.37), and fatigue (p = 0.40). |

Agostini, A. *et al.* (2013) ‘New insights into the brain involvement in patients with Crohn’s disease: a voxel-based morphometry study’, *Neurogastroenterology & Motility*, 25(2), pp. 147–153. doi: 10.1111/nmo.12017.

Agostini, A. *et al.* (2017) ‘Stress and brain functional changes in patients with Crohn’s disease: A functional magnetic resonance imaging study’, *Neurogastroenterology and Motility*, pp. 1–10. doi: 10.1111/nmo.13108.

Bao, C. *et al.* (2017) ‘Differences in brain gray matter volume in patients with Crohn’s disease with and without abdominal pain’, *Oncotarget*, 8(55), pp. 93624–93632. doi: 10.18632/oncotarget.21161.

Bao, C. H. *et al.* (2015) ‘Alterations in brain gray matter structures in patients with Crohn’s disease and their correlation with psychological distress.’, *Journal of Crohn’s & colitis*, 9(7), pp. 532–540. doi: 10.1093/ecco-jcc/jjv057.

Erp, S. van *et al.* (2017) ‘Cerebral magnetic resonance imaging in quiescent Crohn’s disease patients with fatigue’, *World Journal of Gastroenterology*, 23(6), pp. 1018–1029. doi: 10.3748/wjg.v23.i6.1018.

Nair, V. A. *et al.* (2016) ‘Structural imaging changes and behavioral correlates in patients with Crohn’s disease in remission’, *Frontiers in Human Neuroscience*, 10(SEP2016), pp. 1–11. doi: 10.3389/fnhum.2016.00460.

Thomann, A. K. *et al.* (2016) ‘Altered markers of brain development in Crohn’s disease with extraintestinal manifestations - A pilot study’, *PLoS ONE*, 11(9), pp. 1–14. doi: 10.1371/journal.pone.0163202.

Thomann, A. K. *et al.* (2021) ‘Exploring joint patterns of brain structure and function in inflammatory bowel diseases using multimodal data fusion’, *Neurogastroenterology and Motility*, 33(6), pp. 1–10. doi: 10.1111/nmo.14078.

Yeung, A. W. K. (2020) ‘Structural and functional changes in the brain of patients with Crohn’s disease: an activation likelihood estimation meta-analysis’, *Brain Imaging and Behavior*, pp. 1–12. doi: 10.1007/s11682-020-00291-w.

Zikou, A. K. *et al.* (2014) ‘Brain involvement in patients with inflammatory bowel disease: a voxel-based morphometry and diffusion tensor imaging study’, *European Radiology*, (24), pp. 2499–2506. doi: 10.1007/s00330-014-3242-6.

**Figure S1** Consort diagram illustrating the number of participants included in the study


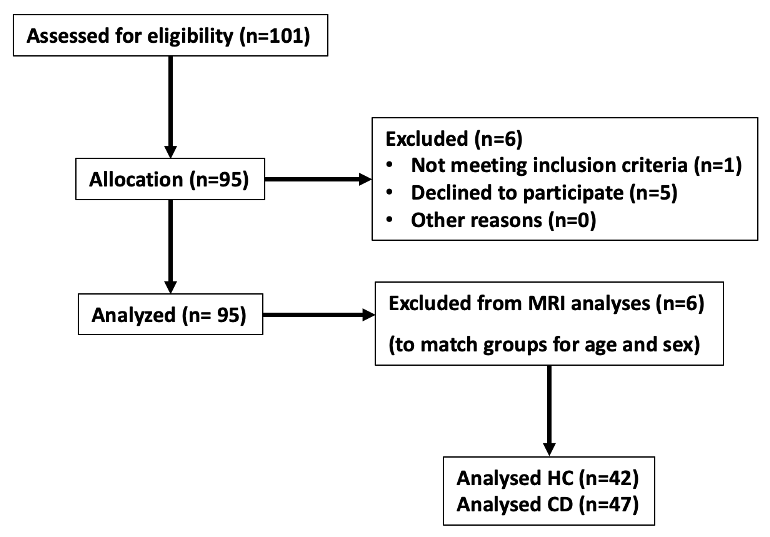


**Table S2** Demographics and clinical characteristics of CD patients and HC. Data provides median and range. (n)= number of subjects, NS = non-significant, NA = not available/missing

|  | **Total CD patients (Active +Remission)**  **(n=47)** | **Active**  **(n=34)** | **Remission**  **(n=13)** | **HC (n=42)** | **Group comparison (p-values)** |
| --- | --- | --- | --- | --- | --- |
| **Age [years]** | 31.0 (18-68) | 30 (18-68) | 44 (23-62) | 30.5 (19-65) | NS |
| **BMI [kg/m2]** | 23.0 (16-34) | 23.6 (16-34) | 23 (19-31) | 24.7 (18.3-31.7) | NS |
| **Sex, n** |  |  |  |  |  |
| **Male** | 26 | 20 | 6 | 23 | NS |
| **Female** | 21 | 14 | 7 | 19 |  |
| **Ethnicity (% Caucasian)** | 42 (89%) | 29 (85%) | 13 (100%) | 35 (84%) | NS |
| **Disease duration [years]** | 7.5 (1- 40) | 5.5 (1-20) | 15 (3-40) | - | P = .006  Active < Remission |
| **C-reactive protein [mg/dl]** | 5 (5-224) | 5.0 (5-224) | 5 (5-5) | - | P = .005  Active >Remission |
| **Faecal calprotectin [µg/g]** | 434 (18-1800) | 434 (18-1800) | NA | - | NS |
| **Harvey Bradshaw index [HBI]** | 3 (0-9) | 3.5 (0-9) | 2 (0-7) | - | Active > Remission  P =.026 |
| **TNF α (pg/ml)** | 3.96 (0 -1234) | 3.96 (0 -1234) | NA | 0 (0-856) | NS |
| **IL-6 (pg/ml)** | 34.8 (0-259) | 34.8 (0-259) | NA | 10.6 (0-492) | NS |
| **IL-1β (pg/ml)** | 1.25 (0 - 1955) | 1.25 (0 - 1955) | NA | 12.17 (0-492) | NS |
| **IBD Fatigue** | 12.0 (0-15) | 12 (3-15) | 10 (0-15) | - | NS |
| **HADS - Anxiety** | 5 (1-11) | 5 (1-11) | 5 (2-11) | 3.5 (0-15) | NS |
| **HADS - Depression** | 3 (0-14) | 3 (0-14) | 3 (0-10) | 1.0 (0-8) | P=.047, Remission>HC |
| **Abdominal pain score** | 2.0 (0-50) | 4 (0-50) | 0 (0-25) | - | NS |
| **CD, (n)** |  |  |  |  |  |
| **with pain** | 27 | 21 | 6 | - | NS |
| **without pain** | 20 | 13 | 7 | - |  |
| **CD, (n)** |  |  |  |  |  |
| **EIM** | 12 | 10 | 2 | - | NS |
| **no EIM** | 35 | 24 | 11 | - |  |
| **Smoking status (n)** |  |  |  |  |  |
| **Never** | 19 | 19 | NA | NA |  |
| **Current** | 2 | 2 | NA | NA |  |
| **Ex-smoker** | 5 | 5 | NA | NA |  |
| **Unknown** | 21 | 21 | NA | NA |  |

**Table S3:** Individual CD patients characteristics with information on treatments, surgery and smoking status.

**A= Active disease, R = remission , M = male , f= Female, EIm = extraintestinal manifestations,ad=adalimumab, inf=infliximab, , MTX=methotrexate, TH = Thiopurine** Montreal classification Age (A); A2=17-40 years, A3= over 40 years. Disease location (L); L1 terminal ileum, L2= colon, L3= ileocolon, L4= upper gastrointestinal tract (a perianal disease modifier may be added in the presence of perianal fistulas). Disease behavior (B); B1= non-stricturing non-penetration, B2= structuring, B3 penetrating

| **ID** | **Disease phase** | **Sex** | **Age (years)** | **BMI (kg/m^2^)** | **Montreal** | **Smoking** | **Medication** | **MRE** | **Colonoscopy** | **disease duration** | **CRP** | **FCP** | **HBI** | **EIM** |
| --- | --- | --- | --- | --- | --- | --- | --- | --- | --- | --- | --- | --- | --- | --- |
| 1 | A | F | 56 | 28 | A3L3B1 | Never | Nil |  | Evidence of inflammatory disease | 1 |  |  | 6 |  |
| 2 | A | M | 18 | 34 | A1L3B2 | Never | INF | Evidence of inflammatory disease |  | 2 | 11 |  | 9 |  |
| 3 | A | M | 31 | 26 | A2L1B1 | Never | Nil |  | Evidence of inflammatory disease | 9 | 24 |  | 6 | erythema nodosum, aphthous ulcers |
| 4 | A | F | 23 | 23 | A2L3B2 | Current | TH, anti-TNF |  | Diffuse punched out ulcerations in distal ileum. | 1 |  |  | 3 |  |
| 5 | A | M | 35 | 34 | A2L1B1 | Never | AD | Evidence of inflammatory disease | Evidence of inflammatory disease | 2 | 52 |  | 0 |  |
| 6 | A | M | 53 | 21 | A2LIB2 | Never | AD, MTX, | Evidence of inflammatory disease |  | 18 | 5 |  | 6 |  |
| 7 | A | M | 30 | 28 | A1L1B3 | Never | TH | Evidence of inflammatory disease |  | 15 | 5 | 30 | 8 |  |
| 8 | A | M | 20 | 19 | A2L1B1 | Never | AZA, |  |  | 3 | 5 | 1027 | 4 |  |
| 9 | A | M | 22 | 24 | A2L1B1 | Never | AZA, | Evidence of inflammatory disease |  | 5 |  |  | 3 |  |
| 10 | A | M | 30 | 29 | A1L1B3 | Never | TH ,INF, |  | Evidence of inflammatory disease | 17 |  |  | 5 | aphthous ulcers |
| 11 | A | M | 44 | 33 | A2L1B2 | Unknown | INF, aminosalicylates | Evidence of inflammatory disease |  | 9 |  |  | 2 | Arthralgia |
| 12 | A | M | 32 | 30 | A2L1B2 | Unknown | TH, AD | Evidence of inflammatory disease |  | 14 |  | 574 | 8 | Arthralgia, erythema nodosum |
| 13 | A | M | 63 | 27 | A3L3B1 | Ex-smoker |  |  | Evidence of inflammatory disease | 20 | 5 | 167 | 3 |  |
| 14 | A | F | 20 | 16 | A2L3B2 | Unknown | NIL |  | Evidence of inflammatory disease | 1 |  |  | 5 | Arthralgia, aphthous ulcers |
| 15 | A | M | 28 | 19 | A2L1B1 | Unknown | Tacrolimus, |  | Evidence of inflammatory disease | 1 | 5 | 174 | 3 |  |
| 16 | A | M | 38 | 31 | A2L1B1 | Ex-smoker | AD, TH |  |  | 7 | 6 | 785 | 8 | uveitis |
| 17 | A | F | 67 | 22 | A3L3B1 | Ex-smoker | MTX, | Evidence of inflammatory disease |  | 1 | 224 | 1800 | 8 | aphthous ulcers, abscess |
| 18 | A | F | 38 | 27 | A2L3B2 | Never | TH | Evidence of inflammatory disease | Evidence of inflammatory disease | 18 | 10 |  | 2 | Abscess |
| 19 | A | M | 68 | 19 | A3L3B1 | Ex-smoker |  |  | Evidence of inflammatory disease. | 1 | 5 | 458 | 9 |  |
| 20 | A | M | 25 | 22 | A1L3B1 | Never | AD | Evidence of inflammatory disease | Evidence of inflammatory disease | 9 | 5 | 607 | 1 |  |
| 21 | A | F | 20 | 20 | A1L3PB1 | Never | INF |  |  | 8 | 25 |  | 0 |  |
| 22 | A | M | 18 | 22 | A1L2B1 | Unknown | TH |  |  | 6 | 5 | 1266 | 8 |  |
| 23 | A | M | 41 | 22 | A2L1B1 | Current | MTX, |  |  | 2 | 5 | 414 | 5 |  |
| 24 | A | F | 19 | 22 | A2L3B3 | Never | NIL |  | Evidence of inflammatory disease | 1 | 11 | 1800 | 1 |  |
| 25 | A | F | 28 | 30 | A2L2B1 | Never | Inf, TH |  |  | 4 | 7 | 319 | 1 |  |
| 26 | A | M | 25 | 30 | A2L3B3 | Never | TH | Evidence of inflammatory disease |  | 8 | 25 | 1800 | 2 |  |
| 27 | A | F | 24 | 22 | A1L2B1 | Never | AD |  | Evidence of inflammatory disease | 11 |  |  | 1 |  |
| 28 | A | F | 62 | 20 | A3L3B1 | Ex-smoker | AD, TH, | Evidence of inflammatory disease |  | 2 | 5 | 404 | 1 |  |
| 29 | A | F | 32 | 29 | A2L3B1 | Never | TH | Evidence of inflammatory disease | Evidence of inflammatory disease | 10 |  |  | 6 | Arthralgia |
| 30 | A | F | 22 | 30 | A2L1B1 | Unknown | AD | Evidence of inflammatory disease | Evidence of inflammatory disease | 2 |  | 30 | 6 | Arthralgia, aphthous ulcers |
| 31 | A | F | 31 | 21 | A2L3B1 | Unknown | Nil | Evidence of inflammatory disease |  | 10 | 5 | 194 | 6 |  |
| 32 | A | F | 24 | 27 | A2L3B1 | Never | TH |  |  | 2 | 5 |  | 3 |  |
| 33 | A | M | 41 | 19 | A2L2B2 | Never | AD, TH |  | Evidence of inflammatory disease | 7 | 5 | 434 | 1 |  |
| 34 | A | M | 18 | 23 | A2L2B1 |  | TH, Inf | Evidence of inflammatory disease |  | 1 | 27 | 18 | 0 |  |
| 35 | R | F | 31 | 27 | A1L2B1 |  | Inf |  |  | 17 | 5 |  | 5 | Uveitis, Aphthous Ulcers, Anal Fissure |
| 36 | R | F | 33 | 31 | A2L2B1 |  | Vedolizumab |  |  | 9 | 5 |  | 4 | Arthralgia |
| 37 | R | M | 52 | 31 | A2L1B3 |  |  |  |  | 18 | 5 |  | 2 |  |
| 38 | R | M | 26 | 19 | A2L2B1 |  |  |  |  | 4 | 5 |  | 1 |  |
| 39 | R | F | 44 | 23 | A2L2B1 |  | Nil |  |  | 17 | 5 |  | 1 |  |
| 40 | R | M | 62 | 27 | A3L1B2 |  |  |  |  |  | 5 |  | 7 |  |
| 41 | R | F | 58 | 23 | A2L2B1 |  |  |  |  | 40 | 5 |  | 2 |  |
| 42 | R | M | 54 | 23 | A2L3B3 |  | AD |  |  | 29 | 5 |  | 0 |  |
| 43 | R | F | 53 | 20 | A3L1B1 |  |  |  |  | 3 | 5 |  | 0 |  |
| 44 | R | M | 23 | 22 | A1L3B1 |  | TH, Inf, |  |  | 8 | 5 |  | 2 |  |
| 45 | R | F | 25 | 23 | A2L2B1 |  | Inf |  |  | 4 | 5 |  | 0 |  |
| 46 | R | F | 54 | 24 | A2L1B2 |  | TH |  |  | 28 | 5 |  | 2 |  |
| 47 | R | M | 26 | 22 | A1L4B1 |  |  |  |  | 13 | 5 |  | 1 |  |

**Table S4:** Global values of TIV, GMV, WMV, CSF and CT (mean ± SEM) in CD and HCs before and after controlling for covariates of no interest. NS – non-significant

|  | CD (n=47) | Active (n=34) | Remission (n=13) | HC  (n=42) | Group comparisons |
| --- | --- | --- | --- | --- | --- |
| **Absolute TIV** | 1394 ± 17.8 | 1386 ± 19.9 | 1416 ± 38.5 | 1431 ± 21.6 | NS |
| **GMV (cm^3^)** |  |  |  |  |  |
| Absolute | 643 ± 10.0 | 645 ± 12.4 | 639 ± 16.9 | 645 ± 10.6 | NS |
| TIV adjusted | 643 ± 5.6 | 648 ± 6.57 | 629 ± 10.6 | 645 ± 5.9 | NS |
| Age, TIV, sex adjusted | 643 ± 3.22 | 644 ± 3.8 | 641 ± 6.3 | 645 ± 3.4 | NS |
| **WMV (cm^3^)** |  |  |  |  |  |
| Absolute | 518 ± 8.8 | 512 ± 9.65 | 535 ± 19 | 526 ± 9.8 | NS |
| TIV adjusted | 518 ± 3.7 | 515 ± 4.3 | 526 ± 7.1 | 526 ± 3.9 | NS |
| Age, TIV, sex adjusted | 518 ± 3.7 | 515 ± 4.4 | 528 ± 7.3 | 527 ± 3.9 | NS |
| **CSF (cm^3^)** |  |  |  |  |  |
| Absolute | 231 ± 5.9 | 227 ± 7.5 | 240 ± 7.9 | 258 ± 8.7 | CD < HC, P=.010,  Active CD < HC, P = .018, Remissive CD < HC; P=.74 |
| TIV adjusted | 230 ± 6.6 | 228 ± 7.8 | 237 ± 12.6 | 258 ± 6.9 | CD < HC, P=.014,  Active CD < HC, P = .014, Remissive CD < HC; P=.45 |
| Age, TIV, sex adjusted | 231 ± 4.9 | 233 ± 5.9 | 225 ± 9.7 | 258 ± 5.3 | CD < HC, P< .001,  Active CD < HC, P = .006, Remissive CD < HC; P=.010 |
| **CT (mm)** |  |  |  |  |  |
| Absolute | 2.63 ± 0.01 | 2.61 ± 0.02 | 2.67 ± 0.03 | 2.63 ± 0.15 | NS |
| Age and sex adjusted | 2.64 ± 0.01 | 2.6 ± 0.01 | 2.7 ± 0.02 | 2.6 ± 0.01 | NS |

**Extended Methods**

1. **Exclusion Criteria**

Exclusion criteria included malignant disease, BMI <18 or > 35 kg/m^2^, significant cardiovascular or respiratory disease, diabetes mellitus, current infection, neurological or cognitive impairment, significant physical disability, significant hepatic disease or renal failure, abnormal blood results other than those explained by CD including bleeding diatheses (in the case of HC all unexplained blood results are exclusion criteria), pregnancy or breastfeeding, severe CD where a delay in a change in medical treatment for 1-2 weeks would not be clinically advisable, or contraindication to MRI (e.g. pacemaker).

1. **Clinical assessments**

The IBD-Fatigue self-assessment scale (first section) was used to identify IBD specific fatigue, its severity, frequency, and duration, where 0 = no fatigue, 1 to 10 = moderate fatigue, 11 to 20 = severe fatigue in both active CD patients and CD in remission(Czuber-Dochan *et al.*, 2014). Abdominal pain was assessed using a 100 mm VAS(Mujagic *et al.*, 2015) with CD patients asked “Do you currently suffer from abdominal (tummy) pain?” yes/no, if yes “how severe is your abdominal (tummy) pain?” on a scale from 0 to 100. The presence or absence of EIM was based on CD participants responses to the “complications” section of the HBI, where CD patients were asked to check boxes that apply (i) none (ii) arthralgia, (iii) uvetis, (iv) erythema nodosum, (v) aphthous ulcers, (vi) pyoderma gangrenosum, (vii) anal fissure, (viii) new fistula and (ix) abscess. Serum levels of interleukin-6 (IL-6), interleukin-1 beta (IL-1 β) and tumour necrosis factor (TNFα) were measured using immunoassay kit (Duoset ELISA Development, R&D Systems, Inc, USA) as previously described(Negm *et al.*, 2019)

Depression and anxiety symptoms were measured by using the Hospital Anxiety and Depression Scale (HADS)(Zigmond and Snaith, 1983), a 14-item questionnaire graded on a 4-point Likert scale with subscales of anxiety and depression, with a sum score ranging from 0 to 21 for each and a cutoff value of >7 on either of the 2 subscales. Scores of 0–7 are considered normal, 8–10 are indicative of mild anxiety/depression symptoms, 11–14 are indicative of moderate anxiety/depression symptoms, and 15–21 are indicative of severe anxiety/depression symptoms(Mikocka-Walus *et al.*, 2016).

1. **Image Pre-processing and Statistical Analysis**

This included bias-field and noise removal, skull stripping, segmentation into grey and white matter, and normalization to MNI space using DARTEL to a 1.5 mm isotropic adult template provided by the CAT12 toolbox. Intensity modulation of the normalized tissue segments accounted for both global affine transformations and local warping. Image data quality was assessed in all individual images from basic image properties, noise and geometric distortions (e.g. due to motion) with a image quality rating (IQR) of 84% (1.5% SD), representing good image quality. Grey and white matter segments were spatially smoothed using a 12 mm FWHM Gaussian smoothing kernel, and edge effects between tissue types avoided by excluding all voxels with values < 0.1 (absolute threshold masking).

For CT analysis, T_1_-weighted images underwent tissue segmentation to estimate white matter distance, i.e. projection-based thickness(Dahnke, Yotter and Gaser, 2013), which handles partial volume information, sulcal blurring, and asymmetries, and topological correction performed based on a spherical harmonics approach (Yotter *et al.*, 2011). Spherical registration was used to adapt the volume-based diffeomorphic DARTEL algorithm to the cortical surface(Ashburner, 2007). Finally, to increase processing speed, all scans were resampled to a higher-resolution 164,000 mesh compatible with FreeSurfer(Fischl, 2012) data, followed by smoothing with a gaussian kernel of 15 mm FWHM.

A region of interest (ROI) analysis was performed for those brain regions that showed significant differences between groups in VBM and CT analysis. The MarsBaR ROI extraction tool (<https://www.fil.ion.ucl.ac.uk/spm/>) was used to extract WMV and GMV values, whereas CT values were extracted using CAT12. The extracted global and regional GMV, WMV and CT values were entered into SPSS Statistics (version 19.0, IBM Corp.) to determine between group differences (active vs remission vs HC) and (all CD vs HC) using an analysis of covariance (ANCOVA) model to control for normalized covariates of no interest such age, TIV and sex. Data were tested for normality using a Shapiro-Wilk test, and since all imaging data were normally distributed values are presented as estimated marginal mean ± SEM. Post-hoc tests were performed with Bonferroni correction for multiple comparisons. Pearson partial correlations were computed to investigate any significant associations between IBDF, abdominal pain and the brain metrics while controlling for age, TIV and sex. Results were considered significant at P<0.05.

Ashburner, J. (2007) ‘A fast diffeomorphic image registration algorithm’, *NeuroImage*, 38(1), pp. 95–113. doi: 10.1016/j.neuroimage.2007.07.007.

Czuber-Dochan, W. *et al.* (2014) ‘Development and psychometric testing of inflammatory bowel disease fatigue (IBD-F) patient self-assessment scale’, *Journal of Crohn’s and Colitis*, 8(11), pp. 1398–1406. doi: 10.1016/j.crohns.2014.04.013.

Dahnke, R., Yotter, R. A. and Gaser, C. (2013) ‘Cortical thickness and central surface estimation’, *NeuroImage*. Elsevier Inc., 65, pp. 336–348. doi: 10.1016/j.neuroimage.2012.09.050.

Fischl, B. (2012) ‘FreeSurfer’, *NeuroImage*, 62(2), pp. 774–781. doi: 10.1016/j.neuroimage.2012.01.021.

Mikocka-Walus, A. *et al.* (2016) ‘Symptoms of Depression and Anxiety Are Independently Associated With Clinical Recurrence of Inflammatory Bowel Disease’, *Clinical Gastroenterology and Hepatology*, 14(6), pp. 829-835.e1. doi: 10.1016/j.cgh.2015.12.045.

Mujagic, Z. *et al.* (2015) ‘Systematic review: Instruments to assess abdominal pain in irritable bowel syndrome’, *Alimentary Pharmacology and Therapeutics*, 42(9), pp. 1064–1081. doi: 10.1111/apt.13378.

Negm, O. H. *et al.* (2019) ‘Patients with tumour necrosis factor (TNF) receptor-associated periodic syndrome (TRAPS) are hypersensitive to Toll-like receptor 9 stimulation’, *Clinical and Experimental Immunology*, 197(3), pp. 352–360. doi: 10.1111/cei.13306.

Yotter, R. A. *et al.* (2011) ‘Topological correction of brain surface meshes using spherical harmonics’, *Human Brain Mapping*, 32(7), pp. 1109–1124. doi: 10.1002/hbm.21095.

Zigmond, A. S. and Snaith, R. P. (1983) ‘The Hospital Anxiety and Depression Scale. Traduction française : J.F. Lépine.’, *Acta Psychiatrica Scandinavica*, 67(6), pp. 361–370. doi: 10.1111/j.1600-0447.1983.tb09716.x.
